# Supplementary material for: Clearing the plate: a strategic approach to mitigate well-to-well contamination in large-scale microbiome studies
Source: mSystems. 2024 Sep 16;9(10):e00985-24. doi: 10.1128/msystems.00985-24 (PMC11494942; doi:10.1128/msystems.00985-24)
Supplement: Supplemental material — Figures S1 and S2, Tables S1 to S4, and supplemental materials and methods. [file msystems.00985-24-s0003.docx]

#### **SUPPLEMENTAL**

**FIGURE S1.** (A) Boxplot showing the DNA yield for each fecal sample measured by fluorescent quantification (PicoGreen, Invitrogen). A Wilcoxon signed-rank test comparing DNA yield from plate-based and Matrix methods are displayed. The black dot depicts the mean. Each point represents a sample and is colored by lab technician. Triangles depict the plate-based method and circles depict the Matrix method. (B-C) Fecal samples from four host subjects separated in principal-coordinate analysis (PCoA) space of unweighted (B) and weighted (C) UniFrac distances calculated using shotgun metagenomics data. Each point is colored by host subject and shaped by extraction method.

**TABLE S1.** Mantel correlations in pairwise distances among samples between the plate-based method and the Matrix Method, for both 16S and metagenomics data. All 16S data were rarefied to 10,000 quality-filtered reads per sample or had samples with fewer than 10,000 reads excluded. All metagenomic data were rarefied to 100,000 quality-filtered reads per sample or had samples with fewer than 100,000 reads excluded. Rarefaction depths were selected to maintain at least 75% of samples.

| Data Type | Distance Metric | n | Pearson's R | p-value |
| --- | --- | --- | --- | --- |
| 16S | Jaccard | 71 | 0.95 | 0.001 |
|  | Canberra | 71 | 0.84 | 0.001 |
|  | Unweighted UniFrac | 71 | 0.94 | 0.001 |
|  | Weighted Unifrac | 71 | 0.77 | 0.001 |
| Metagenomics | Jaccard | 69 | 0.96 | 0.001 |
|  | Canberra | 69 | 0.94 | 0.001 |
|  | Unweighted UniFrac | 69 | 0.95 | 0.001 |
|  | Weighted Unifrac | 69 | 0.79 | 0.001 |

**TABLE S2.** Forward, stepwise regression of factors significantly influencing microbial community beta-diversity for 16S and metagenomics. Values are based on permutation tests of variation explained by redundancy analysis, done separately for unique distance metrics. The full model included extraction method (extraction_protocol) (Plate-based vs Matrix), and host subject identity (host_subject_id) as model variables. Samples were rarefied as described in Table S1.

| 16S | Sample Type | Metric | Significant Variable | R2.adj | Df | AIC | F | Pr(>F) |
| --- | --- | --- | --- | --- | --- | --- | --- | --- |
|  | Human Feces | Jaccard | host_subject_id | 0.99 | 3 | -163.73 | 2557.20 | 0.0002 |
|  |  |  | extraction_protocol | 0.0002 | 1 | -164.75 | 2.75 | 0.04 |
|  |  | Unweighted UniFrac | host_subject_id | 0.99 | 3 | -134.89 | 1237.56 | 0.0002 |
|  |  |  | extraction_protocol | 0.001 | 1 | -136.76 | 3.55 | 0.02 |
|  |  | Weighted UniFrac | host_subject_id | 0.94 | 3 | -64.53 | 203.16 | 0.0002 |
|  |  | RPCA | host_subject_id | 0.81 | 3 | -18.28 | 55.72 | 0.0002 |
|  | Mouse Feces | Jaccard | host_subject_id | 0.96 | 3 | -96.44 | 364.91 | 0.0002 |
|  |  | Unweighted UniFrac | host_subject_id | 0.95 | 3 | -83.78 | 276.88 | 0.0002 |
|  |  | Weighted UniFrac | host_subject_id | 0.64 | 3 | 7.75 | 28.64 | 0.0002 |
|  |  |  | extraction_protocol | 0.03 | 1 | 4.98 | 4.49 | 0.03 |
|  |  | RPCA | host_subject_id | 0.78 | 3 | -15.59 | 55.77 | 0.0002 |
|  | Saliva Before Brushing | Jaccard | host_subject_id | 0.64 | 2 | 5.19 | 23.73 | 0.0002 |
|  |  |  | extraction_protocol | 0.02 | 1 | -19.88 | 3.92 | 0.05 |
|  |  | Unweighted UniFrac | host_subject_id | 0.62 | 2 | 6.12 | 22.51 | 0.0002 |
|  |  |  | extraction_protocol | 0.07 | 1 | -4.65 | 8.18 | 0.003 |
|  |  | Weighted UniFrac | host_subject_id | 0.39 | 2 | 19.03 | 9.39 | 0.0002 |
|  |  | RPCA | host_subject_id | 0.60 | 2 | 7.49 | 20.81 | 0.0002 |
|  | Saliva After Brushing | Jaccard | host_subject_id | 0.64 | 2 | 5.22 | 27.68 | 0.0002 |
|  |  |  | extraction_protocol | 0.05 | 1 | 1.34 | 5.64 | 0.008 |
|  |  | Unweighted UniFrac | host_subject_id | 0.63 | 2 | 6.22 | 26.36 | 0.0002 |
|  |  | Weighted UniFrac | host_subject_id | 0.59 | 2 | 8.91 | 23.00 | 0.0002 |
|  |  | RPCA | host_subject_id | 0.63 | 2 | 5.66 | 27.09 | 0.0002 |
| Shotgun Metagenomics | Sample Type | Metric | Significant Variable | R2.adj | Df | AIC | F | Pr(>F) |
|  | Human Feces | Jaccard | host_subject_id | 0.9 | 3 | -68.10 | 198.15 | 0.0002 |
|  |  | Unweighted UniFrac | host_subject_id | 0.8 | 3 | -9.99 | 47.38 | 0.0002 |
|  |  | Weighted UniFrac | host_subject_id | 0.9 | 3 | -65.63 | 187.28 | 0.0002 |
|  |  |  | extraction_protocol | 0.009 | 1 | -70.78 | 6.90 | 0.006 |
|  |  | RPCA | host_subject_id | 0.7 | 3 | 4.74 | 30.77 | 0.0002 |
|  | Mouse Feces | Jaccard | host_subject_id | 0.7 | 3 | 0.39 | 35.15 | 0.0002 |
|  |  | Unweighted UniFrac | host_subject_id | 0.6 | 3 | 9.34 | 26.57 | 0.0002 |
|  |  | Weighted UniFrac | host_subject_id | 0.5 | 3 | 22.09 | 16.85 | 0.0002 |
|  |  | RPCA | host_subject_id | 0.5 | 3 | 20.41 | 17.98 | 0.0002 |
|  | Saliva Before Brushing | Jaccard | host_subject_id | 0.3 | 2 | 28.45 | 9.29 | 0.0002 |
|  |  | Unweighted UniFrac | host_subject_id | 0.4 | 2 | 23.44 | 13.15 | 0.0002 |
|  |  | Weighted UniFrac | host_subject_id | 0.4 | 2 | 25.76 | 11.29 | 0.0002 |
|  |  | RPCA | host_subject_id | 0.3 | 2 | 31.21 | 7.39 | 0.0002 |
|  | Saliva After Brushing | Jaccard | host_subject_id | 0.5 | 2 | 17.29 | 18.67 | 0.0002 |
|  |  | Unweighted UniFrac | host_subject_id | 0.5 | 2 | 16.35 | 19.60 | 0.0002 |
|  |  | Weighted UniFrac | host_subject_id | 0.5 | 2 | 17.90 | 18.08 | 0.0002 |
|  |  | RPCA | host_subject_id | 0.4 | 2 | 26.33 | 10.86 | 0.0002 |

**TABLE S3.** Mann-Whitney tests comparing alpha diversity between the plate-based method and the Matrix Method for each sample type, using Faith's Phylogenetic Diversity analysis. Samples were rarefied as described in Table S1.

|  | Group 1 | Group 2 | H | p-value | q-value |
| --- | --- | --- | --- | --- | --- |
| Human feces | Plate-based (n=23) | Matrix (n=24) | 0.01 | 0.92 | 0.92 |
| Mouse feces | Plate-based (n=24) | Matrix (n=24) | 1.58 | 0.21 | 0.21 |
| Human saliva | Plate-based (n=36) | Matrix (n=36) | 0.07 | 0.79 | 0.79 |

**FIGURE S2 (A)** Principal component analysis (PCA) of fecal samples color and shape-coded by extraction method and subject. PERMANOVA shows strong separation by subject using both extraction methods (R^2^ = 0.47, F = 8.62, p < 0.001). Asterisks represent centroids of the different factors. **(B)** UpSet plot depicting the intersecting sets of metabolite features detected by each extraction method. More than 75% of the extracted features were recovered by both methods. **(C)** Overlap of discriminating features obtained via pairwise PLS-DA models after 95% ethanol (Matrix Method) or 50% methanol extraction. The underlying pie charts represent percentage of the top 100 discriminating features obtained via the methanol extraction that were also recovered and identified as significant by the Matrix Method.

**Table S3.** Time comparison between the plate-based method and Matrix Method. Plate shaker and SpeedVac^TM^ can process two plates simultaneously. This breakdown

includes multiple KingFisher Flex instruments running simultaneously for both methods. These times represent processing times only, and do not consider the time it takes to walk back and forth from each instrument, as this will vary between each lab.

| **Plate-based Method** | **Process** | **Minutes** | | |
| --- | --- | --- | --- | --- |
|  |  | **1 X 96 well plate** | **2 X 96 well plate** | **4 X 96 well plate** |
|  | Scanning and transferring samples to nucleic acid extraction 96-well plate(s) | 120 | 240 | 480 |
|  | Scanning and transferring samples to metabolite extraction 96-well plate(s) | 120 | 240 | 480 |
|  | Addition of solvent to metabolomics plate(s) | 5 | 5 | 10 |
|  | Shaking of metabolomics plate(s) | 5 | 5 | 10 |
|  | Sonification of metabolomics plate(s) | 5 | 5 | 10 |
|  | Centrifugation of metabolomics plate(s) | 2 | 2 | 2 |
|  | Addition of lysis buffer to nucleic acid extraction plate(s) | 5 | 8 | 15 |
|  | Bead beating of samples in nucleic acid extraction plate(s) | 2 | 2 | 4 |
|  | Centrifugation of nucleic acid extraction plate(s) | 5 | 5 | 5 |
|  | Preparation other reagents e.g. wash buffer | 10 | 15 | 25 |
|  | Transfer of supernatant to 96-well binding extraction plate(s) | 15 | 30 | 60 |
|  | Loading and running of KingFisher(s) - DNA purification instrument | 45 | 50 | 60 |
|  | **TOTAL minutes** | **339** | **607** | **1161** |
|  | **TOTAL hours** | **5.7** | **10.1** | **19.4** |
| **Matrix Method** | **Process** | **Minutes** | | |
|  |  | **1 X 96 well plate** | **2 X 96 well plate** | **4 X 96 well plate** |
|  | Scanning and assembling tubes into matrix rack(s) | 3 | 6 | 12 |
|  | Shaking of matrix rack(s) | 2 | 2 | 4 |
|  | Centrifugation of matrix rack(s) | 5 | 5 | 5 |
|  | Decapping of matrix tubes | 1 | 2 | 4 |
|  | Transfer of supernatant to metabolomics plate(s) | 15 | 30 | 60 |
|  | Sonification of metabolomics plate(s) | 5 | 5 | 10 |
|  | Centrifugation of metabolomics plate(s) | 2 | 2 | 2 |
|  | Vacuum concentration - removing excess storage buffer from matrix tubes | 60 | 60 | 120 |
|  | Addition of beads to matrix tubes | 15 | 30 | 60 |
|  | Addition of lysis buffer to matrix tubes | 5 | 8 | 15 |
|  | Capping of matrix tubes | 1 | 2 | 4 |
|  | Bead beating of samples in matrix rack(s) | 2 | 2 | 4 |
|  | Centrifugation of matrix rack(s) | 5 | 5 | 5 |
|  | Preparation other reagents e.g. wash buffer | 10 | 15 | 25 |
|  | Decapping of matrix tubes | 1 | 2 | 4 |
|  | Transfer of supernatant to 96-well binding extraction plate(s) | 15 | 30 | 60 |
|  | Loading and running of KingFisher(s) - DNA purification instrument | 45 | 50 | 60 |
|  | **TOTAL minutes** | **192** | **256** | **454** |
|  | **TOTAL hours** | **3.2** | **4.3** | **7.6** |
| **Time saved using Matrix Method** |  | **1 X 96 well plate** | **2 X 96 well plate** | **4 X 96 well plate** |
|  | Minutes | 147 | 351 | 707 |
|  | Hours | 2.5 | 5.9 | 11.8 |
|  | % | 43.36 | 57.83 | 60.90 |

#####

**Table S4.** Cost comparison between the plate-based method and Matrix Method.

Consumable and labor cost comparison in USD between the Matrix Method and the plate-based method. All costs are estimates and are subject to change.

| **Matrix Method** | **Consumable/Labor** | **Vendor** | **Catalog Number** | **Amount required per 96 extractions** | **$ per 96 extractions** | **$ per 1 extraction** |
| --- | --- | --- | --- | --- | --- | --- |
|  | Matrix™ ScrewTop Tubes | ThermoFisher Scientific | 3742 | 1 | 74.00 |  |
|  | Matrix™ ScrewTop Tube Caps | ThermoFisher Scientific | 4477 | 1 | 68.00 |  |
|  | Metabolomics Plate | Avantor by VWR | 75870-792 | 1 | 3.50 |  |
|  | Metabolomics Seals | Avantor by VWR | 76196-420 | 1 | 3.35 |  |
|  | Zirconia beads | Fisher Scientific | NC9847287,NC0450473,NC0362415 | 1 | 0.40 |  |
|  | MagMAX™ Lysis Buffer | ThermoFisher Scientific | A42361 | 1 | 41.42 |  |
|  | MagMAX™ Wash Solution | ThermoFisher Scientific | A42360 | 96 mL | 61.42 |  |
|  | MagMAX™ Binding Solution | ThermoFisher Scientific | A42359 | 55 mL | 62.98 |  |
|  | MagMAX™ Binding Beads | ThermoFisher Scientific | A42362 | 2 | 83.80 |  |
|  | MagMAX™ Elution Buffer | ThermoFisher Scientific | A42364 | 6.720 mL | 32.07 |  |
|  | 96-Deep Well Plate | ThermoFisher Scientific | 95040450 | 5 | 33.71 |  |
|  | 96-Deep Well Tip Comb | ThermoFisher Scientific | 97002534 | 1 | 6.66 |  |
|  | EP Motion reservoir 100ml | Eppendorf | 960051017 | 2 | 14.15 |  |
|  | Foil Plate Seals | Eppendorf | 30127889 | 6 | 8.07 |  |
|  | Ethanol | Sigma Aldrich | E7023-4L | 375 mL | 15.00 |  |
|  | KingFisher Elution plate | ThermoFisher Scientific | 97002540 | 2 | 8.01 |  |
|  |  | | | | |  |
|  | Labor |  | | | 70.00 |  |
|  |  | | | **Total** | 586.53 | 6.11 |

| **Plate-based Method** | **Consumable** | **Vendor** | **Catalog Number** | **Amount required per 96 extractions** | **$ per 96 extractions** | **$ per 1 extraction** |
| --- | --- | --- | --- | --- | --- | --- |
|  | MagMAX™™ Microbiome Bead Plate | ThermoFisher Scientific | A42331 | 1 | 157.00 |  |
|  | Metabolomics Plate | Avantor by VWR | 75870-792 | 1 | 3.50 |  |
|  | Metabolomics Seals | Avantor by VWR | 76196-420 | 1 | 3.35 |  |
|  | MagMAX™ Lysis Buffer | ThermoFisher Scientific | A42361 | 1 | 41.42 |  |
|  | MagMAX™ Wash Solution | ThermoFisher Scientific | A42360 | 96 mL | 61.42 |  |
|  | MagMAX™ Binding Solution | ThermoFisher Scientific | A42359 | 55 mL | 62.98 |  |
|  | MagMAX™ Binding Beads | ThermoFisher Scientific | A42362 | 2 | 83.80 |  |
|  | MagMAX™ Elution Buffer | ThermoFisher Scientific | A42364 | 6.720 mL | 32.07 |  |
|  | 96-Deep Well Plate | ThermoFisher Scientific | 95040450 | 5 | 33.71 |  |
|  | 96-Deep Well Tip Comb | ThermoFisher Scientific | 97002534 | 1 | 6.66 |  |
|  | EP Motion reservoir 100ml | Eppendorf | 960051017 | 2 | 14.15 |  |
|  | Foil Plate Seals | Eppendorf | 30127889 | 6 | 8.07 |  |
|  | Ethanol | Sigma Aldrich | E7023-4L | 500 mL | 20.00 |  |
|  | KingFisher Elution plate | ThermoFisher Scientific | 97002540 | 2 | 8.01 |  |
|  | Labor |  | | | 124.30 |  |
|  |  | | | **Total** | 660.43 | 6.88 |

####

##### **MATERIALS AND METHODS**

**Sample Collection**

The sample types, feces and saliva, were used to validate this proposed method due to their importance in human microbiome studies. Human specimens and informed consent were collected under approved protocols from the University of California, San Diego (IRB#150275 for saliva and IRB#141853 for feces). Fecal samples from four volunteers were collected using a commode and promptly stored at -80°C. Human oral samples, from three individuals, were collected by drooling 4 mL into a 50 mL centrifuge tube using a funnel. Mouse feces were collected from four mice using forceps, which were dipped in alcohol and flame sterilized between each collection.

**Well-to-well Contamination**

To assess well-to-well contamination across the plate-based and Matrix Method, four laboratory technicians independently conducted each method in duplicate. Fecal samples were obtained from four volunteers, swabbed in triplicate, and transferred to corresponding positions in both 96-deep well plates and Matrix Tube racks, amounting to a total of 12 fecal swabs per plate and rack. Each swab was surrounded by 84 negative-control extraction blanks (Fig. 1). Both the plate-based and Matrix Method use a 96-sample magnetic bead cleanup format using the KingFisher Flex and the MagMAX^TM^ Microbiome Ultra Nucleic Acid Isolation Kit (catalog # A42357, ThermoFisher, MA, USA).

**DNA Extraction**

Plate-based Method

Wells were taped closed so that only one column at a time was accessible in order to minimize well-to-well contamination during sample loading. DNA extractions were performed following the manufacturer’s instructions (MagMAX^TM^ Microbiome Ultra Nucleic Acid Isolation Kit, catalog # A42357), with lysis performed using a TissueLyser II (Qiagen), and bead clean-ups performed using the automated KingFisher Flex Purification System (ThermoFisher Scientific, MA, USA).

Matrix Method

400 µL of 95% (v/v) ethanol was added to the Matrix Tubes containing samples. Using the automated instrument Capit-All (ThermoFisher Scientific, MA, USA), the tubes were capped and shaken for 2 minutes at 1,200 x g on the SpexMiniG plate shaker, followed by a 5-minute centrifugation at 2,700 x g. Subsequently, using an 8-channel pipette and maintaining sterile technique, the supernatant containing metabolite extract was transferred column by column into a deep well plate suitable for mass spectrometry analysis. This metabolomics plate was then promptly stored at -80°C until further processing. The Matrix Tubes containing the pellet were loaded to a SpeedVac^TM^ at 45°C for 60 min at 5.1 Torr to remove any residual ethanol. 30 µL of 0.1, 0.5 and 1 mm zirconia-silica beads were added to each Matrix Tube using a LabTie bead dispenser (Molgen, Netherlands). 600 µL of Lysis buffer was added to each tube using The Biotek Multiflo bulk reagent dispenser (Agilent, CA, USA). The Matrix Tubes were capped using the Capit-All (ThermoFisher Scientific, MA, USA). Bead beating was performed on the SpexMiniG for 2 min at 1,200 x g. The remaining steps of the DNA extraction followed the manufacturer’s instructions with bead clean-ups performed using the automated KingFisher Flex Purification System (ThermoFisher Scientific, MA, USA). Extracted nucleic acids were stored at -80°C until further processing.

**Quantitative Polymerase Chain Reaction (qPCR)**

The resulting blanks from each extraction were quantified in triplicate reactions via qPCR. The final volume of each qPCR reaction contains 5 μL of SsoAdvanced Sybr Green (2X), 0.5μM of Bakt 341F-805R 16S rRNA gene amplicon primers, 1 µL of sample and 2.5 μL of nuclease free water. Amplification is carried out over 40 cycles (15 s at 95°C, 1 min at 60°C) with an initial 3-minute polymerase activation at 98°C. After amplification a melt curve analysis was performed from 70°C - 98°C at 0.1°C increments per second. Each experiment was performed with a standard curve of bacterial gDNA (ZymoBIOMICS Microbial Community DNA Standard, cat#D6305) consisting of a 10-fold serial dilution (5 ng, 0.5 ng, 0.05 ng, 0.005 ng, 0.0005 ng) in triplicate. Triplicate negative controls containing nuclease free water were also included. The corresponding blank wells from each extraction method were quantified on the same qPCR run to allow for a fair comparison.

**16S AND SHOTGUN METAGENOMICS SEQUENCING**

We prepared DNA for 16S and shotgun metagenomics sequencing as described previously [(1)](https://www.zotero.org/google-docs/?YrBtlf). Metagenomic libraries were normalized by iSeq (Illumina) read count distribution to generate a final pool that made sequencing on the NovaSeq more efficient (2). For 16S data, raw sequence files were demultiplexed using Qiita [(3)](https://www.zotero.org/google-docs/?Yc7xSp), and sub-operational taxonomic units were generated using Deblur [(4)](https://www.zotero.org/google-docs/?JYN20n). For shotgun metagenomics data, raw sequence files were demultiplexed using BaseSpace (Illumina, CA, USA), quality-filtered following previous protocol PMID: 35293792 [(5)](https://www.zotero.org/google-docs/?wxNk1g). Filtered reads were aligned to the Web of Life database [(6)](https://www.zotero.org/google-docs/?kBatyu) using bowtie2 [(7)](https://www.zotero.org/google-docs/?i1C1eX), settings included maximum and minimum mismatch penalties (mp=[1,1]), a penalty for ambiguities (np=1; default), read and reference gap open- and extend penalties (rdg=[0,1], rfg=[0,1]), a minimum alignment score for an alignment to be considered valid (score-min=[L,0,-0.05]), a defined number of distinct, valid alignments (k=16), and the suppression of SAM records for unaligned reads, as well as SAM headers (no-unal, no-hd). Resulting alignments were converted to a feature-table using the Web of Life Toolkit App [(8)](https://www.zotero.org/google-docs/?eDla1F).

**Metabolite Extraction (50% MeOH/H_2_O)**

For comparison to the Matrix Method, replicate fecal samples were weighed and 50% MeOH/H_2_O was added at a ratio of 20 mg:800 µL (sample:solvent). Then, the samples were homogenized using a single 5 mm stainless steel bead in a TissueLyser II (QIAGEN) for 5 min at 25 Hz. Samples were incubated at 4°C for 30 min, dried overnight in a CentriVap and stored at -80°C until resuspension.

The samples were resuspended with 200 µL of 50% MeOH/H_2_O containing 1 µM of sulfadimethoxine as internal standard followed by incubation at -20°C for 1 h. Lastly, the samples were centrifuged at 21,130 x g for 10 min and the supernatant was transferred to a 2 mL glass vial (Thermo Scientific) for LC-MS/MS analysis.

**LC-MS/MS DATA ACQUISITION**

Untargeted metabolomics analysis was performed using a Vanquish UHPLC system (ultra-high performance liquid chromatography) coupled to a Q-Exactive Orbitrap mass spectrometer (Thermo Fisher Scientific). A C18 column (1.7 µm particle size, 2.1 mm x 50 mm Phenomenex, Kinetex) was used for chromatography with a flow rate of 0.5 mL/min. The mobile phase consisted of solvent A (water + 0.1% formic acid) and solvent B (ACN + 0.1% formic acid). Five microlitres of samples was injected and eluted using the following gradient: 0–1 min 5% B, 1–7 min 5 – 99% B, 7–8 min 99% B, 8–8.5 min 99 –5% B, 8.5–10 min 5% B. Mass spectrometry (MS) data was acquired in data-dependent acquisition mode with positive ionization using electrospray ionization (ESI). ESI parameters were set to 53 L/min sheath gas flow, 14 L/min aux gas flow rate, 3 L/min sweep gas flow, 3.5 kV spray voltage, 269°C intel capillary, and aux gas heater set to 438°C. MS scan range was set to 100 – 1500 m/z with a resolution at m/z 200 set to 35,000 with 1 microscans. Automatic gain control (AGC) was set to 5E4 with a maximum injection time of 50 ms. Up to 5 MS/MS (TopN = 5) spectra per MS1 were collected with a resolution at m/z 200 set to 17,500 with 1 microscans. Injection time was set to 50 ms with an AGC target of 5E4. The isolation window was set to 2.0 m/z. Normalized collision energy was set to a stepwise increase of 20, 30, and 40 with an apex trigger set to 2 –15 s and a dynamic exclusion of 10 s. Isotope peaks were excluded.

**LC-MS/MS DATA PROCESSING AND ANALYSIS**

Raw data were converted into .mzML open-access using ProteoWizard msConvert (9). Feature detection and extraction were performed using batch processing in MZmine 3.9 (10). Processing parameters can be found in the .xml file deposited, together with the raw data, in the MSV000095260 public dataset. Obtained feature table and .mgf file were imported into GNPS ([https://gnps.ucsd.edu/ProteoSAFe/status.jsp?task=bf5f87a1db834a608ac7bfdc61eb0b80)](https://gnps.ucsd.edu/ProteoSAFe/status.jsp?task=bf5f87a1db834a608ac7bfdc61eb0b80) for FBMN (11). Feature table was then imported in R 4.2.2 (The R Foundation for Statistical Computing, Vienna, Austria) for multivariate analyses. Before dimensionality reduction, data were cleaned via blank subtraction and via the use of quality control samples. Principal component analysis (PCA) and partial least square - discriminant analysis (PLS-DA) were performed on the robust center log ratio transformed table via mixOmics v 6.22 package (12). Model performances were evaluated using leave one out (loo) cross-validation. PERMANOVA on the Aitchison distances was calculated using the vegan v 2.6 package. The Upset plot was generated using the UpSetR v 1.4 package. Features discriminating the different subjects were obtained via pairwise PLS-DA models per extraction method. Features were considered significant if they had a VIP score > 1. Obtained significant features from the 50% methanol and 95% ethanol extraction methods were then intersected to identify overlapping ones and to check how many of the top 100 most important features for the 50% methanol method were also recovered and significant by the 95% ethanol one.

#### **SUPPLEMENTAL REFERENCES**

1. Sanders JG, Nurk S, Salido RA, Minich J, Xu ZZ, Zhu Q, et al. Optimizing sequencing protocols for leaderboard metagenomics by combining long and short reads. Genome Biol. 2019 Dec;20(1):226.
2. Brennan C, Salido RA, Belda-Ferre P, Bryant M, Cowart C, Tiu MD, et al. Maximizing the potential of high-throughput next-generation sequencing through precise normalization based on read count distribution. Sachdeva N, editor. mSystems. 2023 Jun 23;e00006-23.
3. Gonzalez A, Navas-Molina JA, Kosciolek T, McDonald D, Vázquez-Baeza Y, Ackermann G, et al. Qiita: rapid, web-enabled microbiome meta-analysis. Nat Methods. 2018 Oct;15(10):796–8.
4. Amir A, McDonald D, Navas-Molina JA, Kopylova E, Morton JT, Zech Xu Z, et al. Deblur Rapidly Resolves Single-Nucleotide Community Sequence Patterns. Gilbert JA, editor. mSystems. 2017 Apr 21;2(2):e00191-16.
5. Armstrong G, Martino C, Morris J, Khaleghi B, Kang J, DeReus J, et al. Swapping Metagenomics Preprocessing Pipeline Components Offers Speed and Sensitivity Increases. mSystems. 2022 Apr 26;7(2):e0137821.
6. Zhu Q, Mai U, Pfeiffer W, Janssen S, Asnicar F, Sanders JG, et al. Phylogenomics of 10,575 genomes reveals evolutionary proximity between domains Bacteria and Archaea. Nat Commun. 2019 Dec;10(1):5477.
7. Langmead B, Salzberg SL. Fast gapped-read alignment with Bowtie 2. Nat Methods. 2012 Apr;9(4):357–9.
8. Zhu Q, Huang S, Gonzalez A, McGrath I, McDonald D, Haiminen N, et al. Phylogeny-Aware Analysis of Metagenome Community Ecology Based on Matched Reference Genomes while Bypassing Taxonomy. Sharpton TJ, editor. mSystems. 2022 Apr 26;7(2):e00167-22.
9. Chambers MC, Maclean B, Burke R, Amodei D, Ruderman DL, Neumann S, et al. A cross-platform toolkit for mass spectrometry and proteomics. Nat Biotechnol. 2012 Oct;30(10):918–20.
10. Schmid R, Heuckeroth S, Korf A, Smirnov A, Myers O, Dyrlund TS, et al. Integrative analysis of multimodal mass spectrometry data in MZmine 3. Nat Biotechnol. 2023 Apr;41(4):447-449. doi: 10.1038/s41587-023-01690-2. PMID: 36859716; PMCID: PMC10496610.
11. Nothias LF, Petras D, Schmid R, Dührkop K, Rainer J, Sarvepalli A, et al. Feature-based molecular networking in the GNPS analysis environment. Nat Methods. 2020 Sep;17(9):905–8.
12. Rohart F, Gautier B, Singh A, Lê Cao KA. mixOmics: An R package for ‘omics feature selection and multiple data integration. Schneidman D, editor. PLoS Comput Biol. 2017 Nov 3;13(11):e1005752.
